# Electronic properties of a two-dimensional van der Waals MoGe_2_N_4_/MoSi_2_N_4_ heterobilayer: effect of the insertion of a graphene layer and interlayer coupling

**DOI:** 10.1039/d1ra04531h

**Published:** 2021-08-25

**Authors:** D. K. Pham

**Affiliations:** Institute of Applied Technology, Thu Dau Mot University Binh Duong Province Vietnam phamdinhkhang@tdmu.edu.vn

## Abstract

van der Waals heterostructures (vdWHs) based on 2D layered materials with select properties are paving the way to integration at the atomic scale, and may give rise to new heterostructures exhibiting absolutely novel physics and versatility. Herein, we investigate the structural and contact types in a 2D vdW heterobilayer between MoGe_2_N_4_ and MoSi_2_N_4_ monolayers, and the monolayers in the presence of electrical graphene (GR) contact. In the ground state, the MoGe_2_N_4_/MoSi_2_N_4_ heterobilayer forms type-II band alignment, which effectively promotes the separation of electrons and holes and provides opportunity for further electrons and holes. Thus, the MoGe_2_N_4_/MoSi_2_N_4_ heterobilayer is promising for designing optoelectronic devices with significantly suppressed carrier-recombination. Interestingly, the insertion of the GR contact to a MoGe_2_N_4_/MoSi_2_N_4_ heterobilayer gives rise to the formation of a metal/semiconductor contact. Depending on the GR position relative to the MoGe_2_N_4_/MoSi_2_N_4_ heterobilayer, the GR-based heterostructure can form either an n-type or p-type Schottky contact. Intriguingly, the contact barriers in the GR contacted MoGe_2_N_4_/MoSi_2_N_4_ heterobilayer are significantly smaller than those in the GR contacted with MoGe_2_N_4_ or MoSi_2_N_4_ monolayers, suggesting that the GR/MoGe_2_N_4_/MoSi_2_N_4_ heterostructure offers an effective pathway to reduce the Schottky barrier, which is highly beneficial for improving the charge injection efficiency of the contact heterostructures. More interestingly, by controlling the interlayer coupling through stacking, both the Schottky barriers and contact types in the GR/MoGe_2_N_4_/MoSi_2_N_4_ heterostructure can be manipulated. Our findings could provide theoretical insight into the design of nanodevices based on a GR and MoGe_2_N_4_/MoSi_2_N_4_ heterobilayer.

## Abstract 2

We investigate the structural and contact types in a 2D van der Waals heterobilayer between MoGe_2_N_4_ and MoSi_2_N_4_ monolayers, and the monolayers in the presence of electrical graphene.

## Graphical abstract

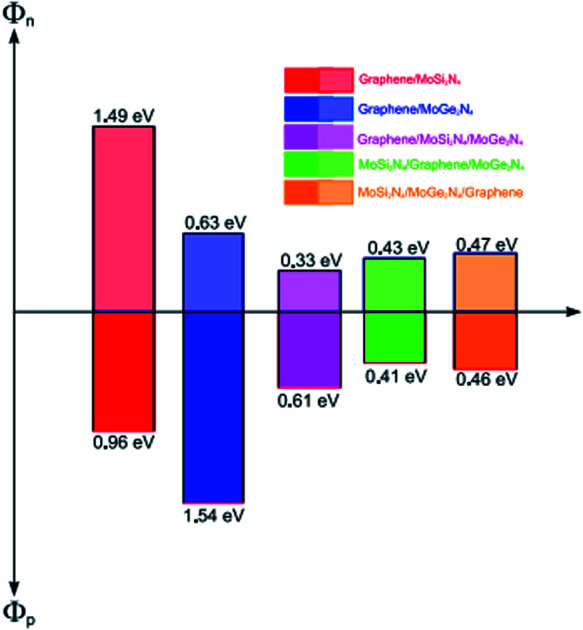

## 1. Introduction

Two-dimensional (2D) materials^1–3^ have received extensive interest owing to their intriguing physical and chemical properties and broad device application potential, including in electronics,^3,4^ nanophotonics,^5^ optoelectronics^6,7^ and spintronics.^8,9^ A plethora of 2D materials, including graphene (GR),^10^ phosphorene,^11,12^ transition metal dichalcogenides (TMDCs),^13,14^ and graphitic carbon nitrides,^15–17^ have been synthesized experimentally and predicted theoretically. These 2D materials exhibit outstanding properties and unique advantages over conventional bulk materials, and are particularly promising candidates for designing high-performance nanodevices, such as field-effect transistors^18^ and photodetectors.^19^

Very recently, Hong *et al.*^20^ discovered a new family of 2D materials, namely MoSi_2_N_4_ monolayers, using chemical vapor deposition (CVD). The MoSi_2_N_4_ monolayer is a layered structure, which can be viewed as a MoN_2_ layer sandwiched between two Si–N bilayers. The MoSi_2_N_4_ monolayer exhibits semiconducting characteristics with a band gap of about 1.94 eV, and possesses high carrier mobility up to 1200 cm^2^ V^−1^ s^−1^. This monolayer is also stable and mechanically stronger than most other 2D semiconductors such as MoS_2_ monolayers. The successful synthesis of MoSi_2_N_4_ monolayers has led to a new class of 2D materials with formula MA_2_Z_4_,^20^ where M represents an early transition metal (Mo, W, Cr), A = Si or Ge, and Z = N, P, or As. Motivated by this finding, many theoretical investigations have been performed to explore the electronic, optical and transport properties of MA_2_Z_4_ monolayers. For instance, Guo *et al.*^21^ studied the electronic properties and transport coefficient of MoSi_2_N_4_ monolayers alongside the strain effect, and showed that band gap and Seebeck coefficient of such a monolayer are strain-tunable. Wu and colleagues^22^ also predicted that the band gap of MSi_2_N_4_ (M = Mo, W) monolayers can be altered by strain and electric field, which result in the transformation from indirect to direct band gap semiconductors. Furthermore, MoSi_2_N_4_, WSi_2_N_4_ and VSi_2_N_4_ monolayers are also predicted to be a potential valleytronic materials.^23,24^ These ever expanding first-principle calculations of MA_2_Z_4_ have continually revealed the enormous potential of MA_2_Z_4_ monolayers for future high-performance device applications.

Currently, constructing 2D van der Waals (vdW) heterostructures between two or more 2D materials is known to be an effective strategy to explore more new properties and extend the potential applications of the corresponding 2D materials. These 2D vdW heterostructures have been predicted theoretically using first-principles calculations, and synthesized experimentally *via* several methods, such as CVD and mechanical exfoliation. In addition, 2D vdW heterostructures have new desirable properties which could be used for fabricating high-efficiency nanodevices experimentally, such as for solar cells, field-effect transistors (FFTs), and photodetectors. Recently, the combination between the layers of several MA_2_Z_4_ monolayers to form MA_2_Z_4_ bilayers,^22,25^ and between the GR layer and MA_2_Z_4_ monolayers,^26,27^ has received much attention from the scientific community. For instance, Zhong *et al.*^25^ investigated the electronic features of MA_2_Z_4_ (M = Ti, Cr, Mo; A = Si and Z = N, P) bilayers under the strain effect. They predicted that the electronic properties of the MA_2_Z_4_ family are tunable with strain-induced transitions from semiconductor to metal. This finding makes the MA_2_Z_4_ family a potential candidate for fabricating electro-mechanical devices. Similarly, Wu and colleagues^22^ showed that applying an electric field can also tune the semiconductor-to-metal transition in both MoSi_2_N_4_ and WSi_2_N_4_ bilayers, they are therefore suitable for designing next generation nanoelectronics and optoelectronics. Nonetheless, the combination between MoSi_2_N_4_ and MoGe_2_N_4_ to form a heterobilayer, and between graphene and MoSi_2_N_4_/MoGe_2_N_4_ heterobilayer to form a GR/MoSi_2_N_4_/MoGe_2_N_4_ heterostructure, have not yet been explored thoroughly.

In this work, based on first-principles calculations, we investigate the electronic structures of the MoSi_2_N_4_/MoGe_2_N_4_ heterobilayer and explore its electronic properties and band alignment alongside the presence of the GR layer. Various combinations and stacking configurations of GR/MoSi_2_N_4_/MoGe_2_N_4_ heterostructures are investigated. Our results reveal that the MoSi_2_N_4_/MoGe_2_N_4_ heterobilayer exhibits an indirect band gap semiconductor and possesses type-II band alignment at the equilibrium state. With the presence of the GR layer in the MoSi_2_N_4_/MoGe_2_N_4_ heterobilayer, we see the formation of the Schottky contact with narrow barrier height of about 0.26 eV, which increases the carrier injection efficiency.

## 2. Computational details

In this work, all the calculations, including geometric optimization, electronic properties and band alignment of the MoSi_2_N_4_/MoGe_2_N_4_ heterobilayers and the heterostructures between GR and MoSi_2_N_4_/MoGe_2_N_4_ heterobilayer, were performed from first-principles calculations within density functional theory, which was implemented in Vienna *Ab initio* Simulation (VASP)^28^ and Quantum Espresso^29,30^ packages. The Perdew–Burke–Ernzerhof (PBE) in the framework of the generalized gradient approximation (GGA)^31^ was used to describe the exchange–correlation energy. The projector augmented wave (PAW)^32^ pseudopotentials were used to describe the electron-ion interaction. In addition, the DFT-D3 method of Grimme^33^ was also selected for describing the presence of the weak vdW forces that always occur in layered vdW systems, including heterobilayers and heterostructures. Furthermore, it should be noted that the traditional DFT method always underestimates the band gap values of materials, especially 2D materials, we therefore used the Heyd–Scuseria–Ernzerhof (HSE06) hybrid functional to avoid this issue and obtain an accurate band gap of the considered materials. The Brillouin zone (BZ) was sampled using the Monkhorst-Pack scheme. A 12 × 12 × 1 (6 × 6 × 1) *k*-point mesh was used for all the calculations within the DFT-PBE (HSE06) method with a cutoff energy of 510 eV. To avoid the interactions between periodical slabs, we set a large vacuum thickness of 25 Å along the *z* direction. The geometric optimization was fully obtained with the convergence threshold for energy of 10^−6^ eV and for a force of 10^−3^ eV Å^−1^. The spin–orbit coupling (SOC) was also taken into account for the electronic properties of the heterobilayer.

## 3. Results and discussion

### 3.1. Structural and electronic properties of MoGe_2_N_4_/MoSi_2_N_4_ heterobilayer

**Fig. 1:**
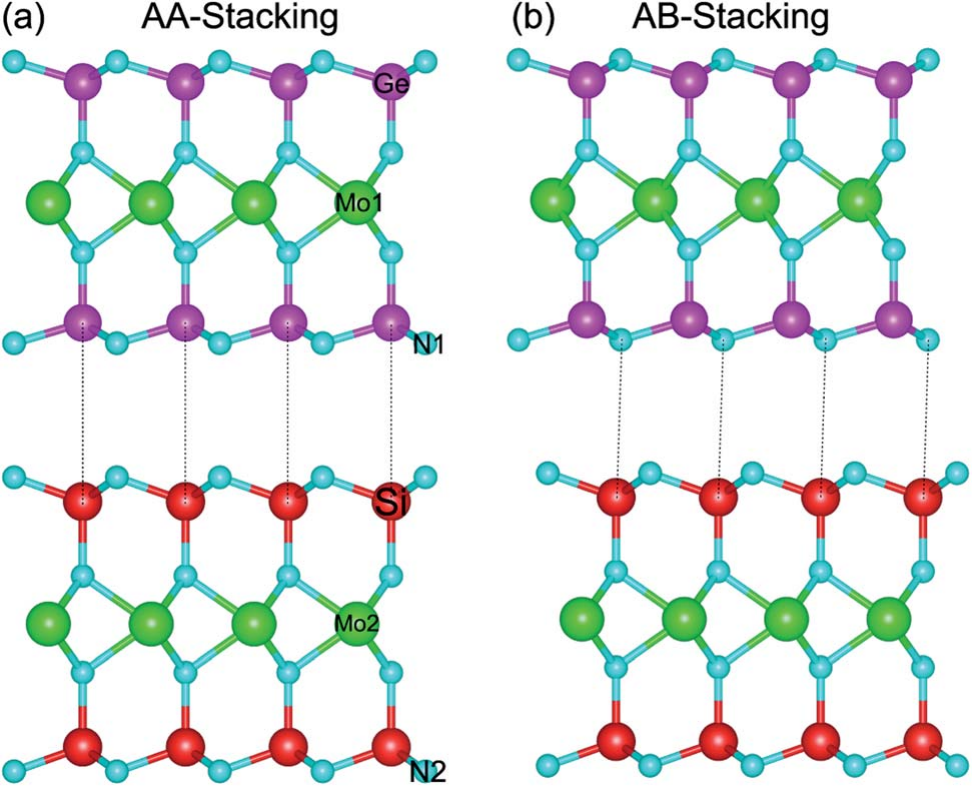
Side view of the optimized atomic structures of the MoGe_2_N_4_/MoSi_2_N_4_ heterostructure for (a) AA-stacking and (b) AB-stacking.

The relaxed atomic structures of the MoGe_2_N_4_/MoSi_2_N_4_ heterostructure for AA and AB stacking configurations are depicted in Fig. 1. In AA-stacking, both the Ge and N atoms in the MoGe_2_N_4_ layer are located directly above the Si and N atoms of the MoSi_2_N_4_ layer, as depicted in Fig. 1(a). Whereas, in AB-stacking, the Ge and N atoms in the MoGe_2_N_4_ layer are staggered with respect to the Si and N atoms of the MoSi_2_N_4_ layer, as depicted in Fig. 1(b). After geometric optimization, the interlayer spacings (*D*) between the MoGe_2_N_4_ and MoSi_2_N_4_ layers for the AA and AB stacking configurations are obtained as listed in Table 1. For the AA-stacking, the equilibrium interlayer spacing is 3.33 Å, and it is 3.35 Å for the AB-stacking configuration. For checking the structural stability of such a heterostructure, we further calculate the binding energy as: *E*_b_ = *E*_H_ − Σ*E*_Mi_, where *E*_H_ is the total energy of the combined MoGe_2_N_4_/MoSi_2_N_4_ heterostructure. *E*_Mi_ is the total energy of the constituent MoGe_2_N_4_ and MoSi_2_N_4_ monolayers. The binding energy of the MoGe_2_N_4_/MoSi_2_N_4_ heterobilayer for AA and AB stacking configurations is calculated to be −0.11 and −0.16 eV, respectively. The negative binding energy confirms that the heterobilayer is dynamically stable. In addition, the binding energy of the MoGe_2_N_4_/MoSi_2_N_4_ heterobilayer for the AB stacking configuration is lower than that for the AA stacking configuration, suggesting that the AB stacking configuration can be considered as the most energetically favorable stacking configuration.

**Table 1:** Optimized lattice constant *a* (Å), equilibrium interlayer spacing *D* (Å), binding energy *E*_b_ (eV), band gap *E*_g_ (eV) obtained from PBE, HSE and PBE + SOC and band alignment of MoGe_2_N_4_/MoSi_2_N_4_ heterostructures for different stacking configurations

	*a*	*D*	*E*_b_	*E*_g_			Band alignment
				PBE	HSE	PBE + SOC	
AA-stacking	2.96	3.33	−0.11	0.88	1.29	0.87	Type-II
AB-stacking	2.96	3.35	−0.16	0.90	1.33	0.89	Type-II

**Fig. 2:**
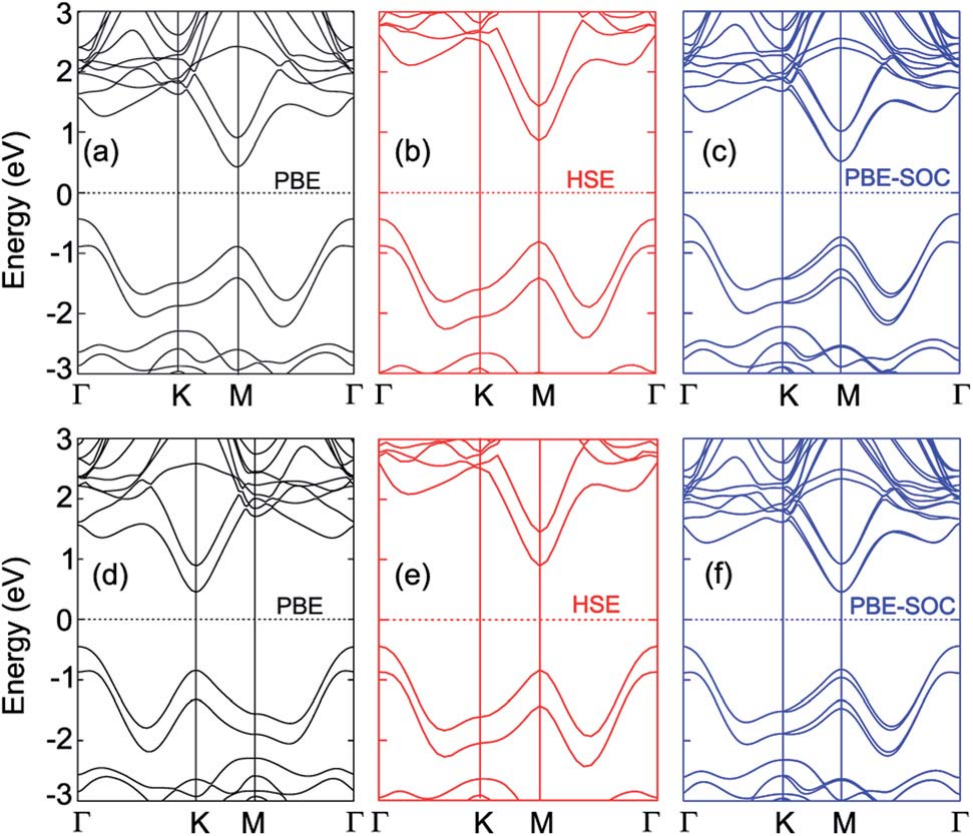
Band structures of the MoGe_2_N_4_/MoSi_2_N_4_ heterostructure calculated by (a, d) PBE, (b, e) HSE and (c, f) PBE + SOC methods for (a–c) AA-stacking and (d–f) AB-stacking.

The band structures of the MoGe_2_N_4_/MoSi_2_N_4_ heterostructure for different stacking configurations are displayed in Fig. 2. We used different methods, including PBE, HSE and PBE + SOC to calculate the band gaps of the considered heterostructure. The obtained band gaps for the MoGe_2_N_4_/MoSi_2_N_4_ heterostructure are listed in Table 1. For the AA-stacking, the band gaps of the MoGe_2_N_4_/MoSi_2_N_4_ heterostructure are calculated as 0.88 eV, 1.29 eV and 0.87 eV. It is clear that the HSE06 method gives rise to a larger band gap value than the PBE and PBE + SOC methods. However, all three methods predict the same characters of the MoGe_2_N_4_/MoSi_2_N_4_ heterostructure, which possesses the indirect band gap semiconductor. The valence band maximum (VBM) is located at the *Γ* point, while the conduction band minimum (CBM) is located at the *M* point. Interestingly, when the SOC effect is applied, it tends to split the valence bands at the *M* point of the MoGe_2_N_4_/MoSi_2_N_4_ heterostructure into two different parts, as depicted in Fig. 2(c). The energy of band splitting for the first and second valence bands of the AA-stacking is calculated to be 13.27 meV and 13.9 meV, respectively. However, the SOC effect splits only the valence bands at the *M* point, whereas the VBM of such heterostructure is located at the *Γ* point. Therefore, the SOC does not affect the band alignments of the heterostructures. Similar trends are also observed in the AB-stacking of the MoGe_2_N_4_/MoSi_2_N_4_ heterostructure. The PBE, HSE06 and PBE + SOC band gaps of the MoGe_2_N_4_/MoSi_2_N_4_ heterostructure for the AB-stacking are 0.90, 1.33 and 0.89 eV, respectively. The AB-stacking also predicts the indirect band gap semiconductor in the MoGe_2_N_4_/MoSi_2_N_4_ heterostructure with the VBM at the *Γ* point and the CBM at the *M* point, as depicted in Fig. 2(d–f). Furthermore, the band offset is also critical for the optoelectronic application, we therefore calculate the band offsets for valence bands (*Δ*_V_) and for conduction bands (*Δ*_C_). The band offsets *Δ*_V_ and *Δ*_C_ for the AA-stacking are calculated to be 0.43 eV and 0.47 eV, respectively. Whereas, these values for AB-stacking are 0.40 eV and 0.43 eV, respectively. In addition, comparing AA and AB stacking, the band structure and alignment seems to be almost unchanged. This is a very convenient behaviour for experimental fabrication since the electronic properties are less affected by stacking configurations.

**Fig. 3:**
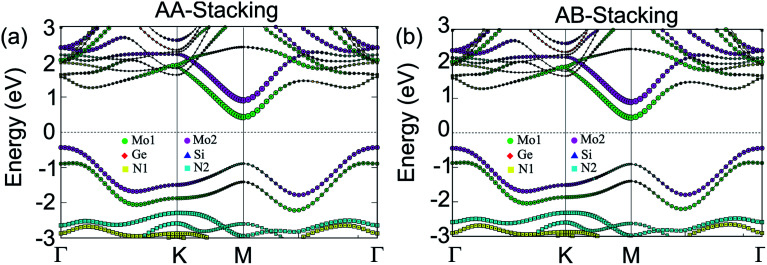
Weighted band structures of the MoGe_2_N_4_/MoSi_2_N_4_ heterostructure for (a) AA-stacking and (b) AB-stacking.

More interestingly, device performance based on vdW heterostructures depends crucially on the band alignment, forming between two different 2D materials. Depending on the band edge positions of the constituent monolayers, vdW heterostructure can be divided into three different types of band alignment, including type-I (straddling gap), type-II (staggered gap) and type-III (broken gap). To investigate the band alignment, we further calculate weighted band structures of the MoGe_2_N_4_/MoSi_2_N_4_ heterostructure, as depicted in Fig. 3. For both the AA-stacking and AB-stacking, one can find that the CBM of the MoGe_2_N_4_/MoSi_2_N_4_ heterostructure is mainly contributed by the Mo-orbital states of the MoGe_2_N_4_ layer, as marked with green circles. Whereas, the VBM at the *Γ* point is mainly contributed by the Mo-orbital states of the MoSi_2_N_4_ layer, as marked by the violet circles. The contributions by the different monolayers to the VBM and CBM demonstrate that the MoGe_2_N_4_/MoSi_2_N_4_ heterostructure possesses type-II band alignment for both AA- and AB-stacking. The type-II band alignment promotes effective separation of electrons and holes and provides an opportunity for electrons and holes to separate in real space. Thus, the MoGe_2_N_4_/MoSi_2_N_4_ heterostructure is promising for designing optoelectronic devices with significantly suppressed carrier-recombination.

**Fig. 4:**
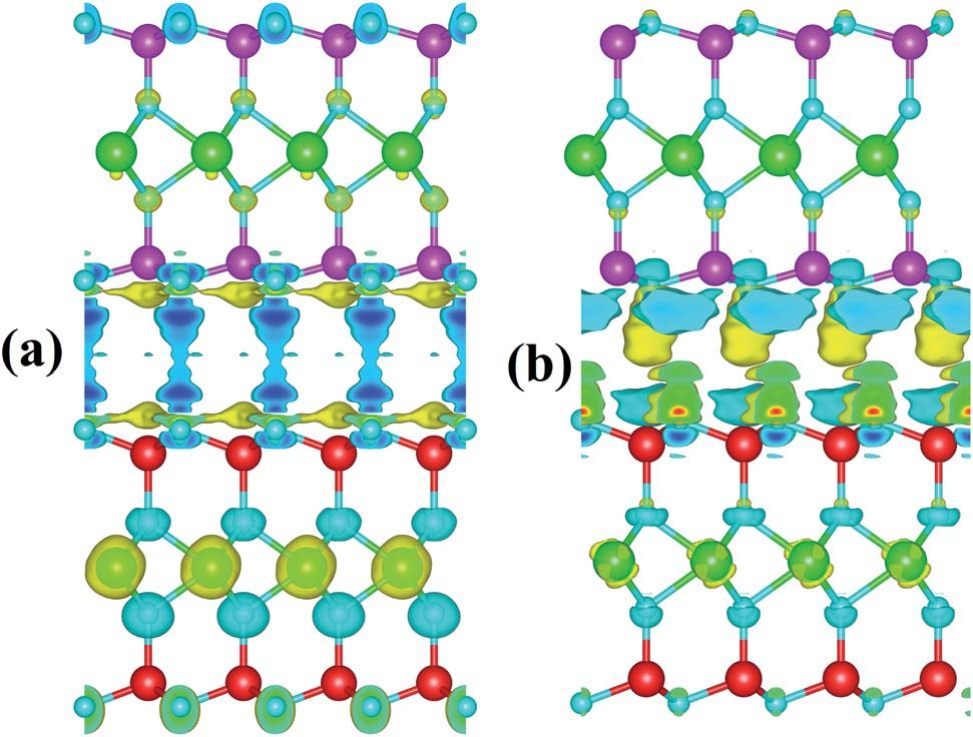
Charge density difference of the MoGe_2_N_4_/MoSi_2_N_4_ heterostructure for the (a) AA- and (b) AB-stacking configurations. Yellow and cyan regions represent the positive and negative charges, respectively. The isosurface value is set to be 2.35 e Å^−3^.

We further investigated the charge mechanism in the MoGe_2_N_4_/MoSi_2_N_4_ heterobilayer by calculating the charge density difference as follows: Δ*ρ* = *ρ*_HB_ − Σ*ρ*_Mi_, where *ρ*_HB_ and *ρ*_Mi_, respectively, are the charge density of the MoGe_2_N_4_/MoSi_2_N_4_ heterobilayer and the constituent MoGe_2_N_4_ and MoSi_2_N_4_ monolayers. The charge density difference of the MoGe_2_N_4_/MoSi_2_N_4_ heterobilayer for the AA- and AB stacking is illustrated in Fig. 4. The yellow and cyan regions represent the positive and negative charges, respectively. We find that the charges are mainly visualized at the interface. The yellow regions are located in the MoGe_2_N_4_ side, while the cyan regions are in the MoSi_2_N_4_ layer. This finding suggests that the charges are mainly accumulated in the MoGe_2_N_4_ layer and depleted in the MoSi_2_N_4_ layer.

### 3.2. Electrical graphene contact to a MoGe_2_N_4_/MoSi_2_N_4_ heterobilayer

**Fig. 5:**
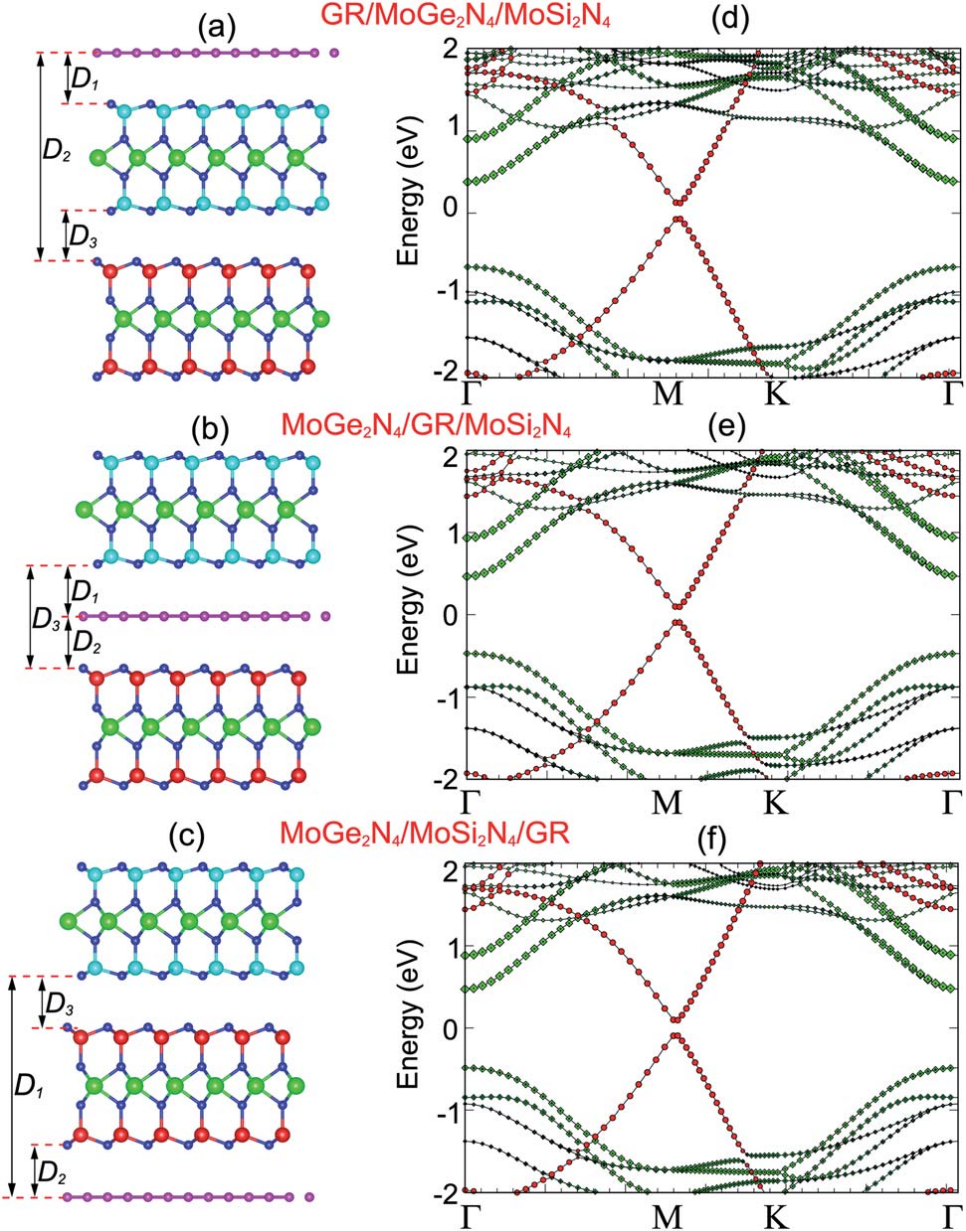
Side view of the atomic structures of (a) GR/MoGe_2_N_4_/MoSi_2_N_4_, (b) MoGe_2_N_4_/GR/MoSi_2_N_4_ and (c) MoGe_2_N_4_/MoSi_2_N_4_/GR heterostructures. Weighted band structures of (d) GR/MoGe_2_N_4_/MoSi_2_N_4_, (e) MoGe_2_N_4_/GR/MoSi_2_N_4_ and (f) MoGe_2_N_4_/MoSi_2_N_4_/GR heterostructures. Red and green circles represent the contributions of semimetal graphene and semiconducting MoGe_2_N_4_/MoSi_2_N_4_ layers, respectively.

**Fig. 6:**
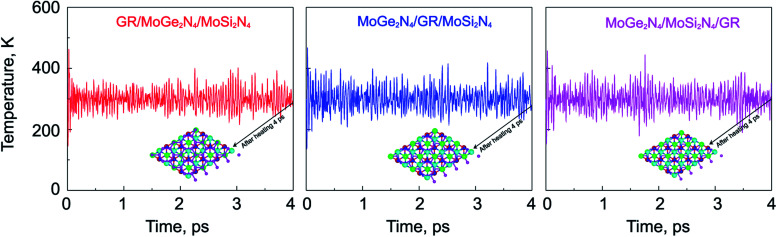
The variation of the temperature of the considered heterostructures as a function of a time heating step by performing AIMD simulations. The insets represent snapshots of the heterostructures after heating for 4 ps.

We now consider the formation of a triple-layered 2D-metal/2D-semiconductor heterostructure by including a graphene layer into the MoSi_2_N_4_/MoGe_2_N_4_ heterobilayer. The atomic structures of all vdW heterostructures between the GR and MoSi_2_N_4_/MoGe_2_N_4_ heterobilayer are depicted in Fig. 5(a–c). Three stacking sequences can be obtained: GR layer placed on top of the MoSi_2_N_4_ layer in the MoSi_2_N_4_/MoGe_2_N_4_ heterobilayer (Fig. 5(a)); GR layer between the MoSi_2_N_4_ and MoGe_2_N_4_ layers (Fig. 5(b)); and GR layer located below the MoGe_2_N_4_ layer in the MoSi_2_N_4_/MoGe_2_N_4_ heterobilayer (Fig. 5(c)). It is clear that the lattice constant of single-layer GR is calculated to be 2.46 Å, while the lattice constant of the MoSi_2_N_4_/MoGe_2_N_4_ heterobilayer is calculated to be 2.98 Å. Therefore, in order to avoid the effect of lattice mismatch, we use a supercell, consisting of (2 × 2) unit cells of GR and 
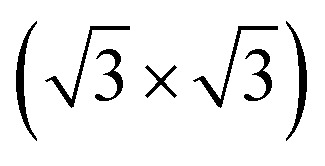
 unit cells of MoSi_2_N_4_/MoGe_2_N_4_ heterobilayer to build the triple-layered 2D-metal/2D-semiconductors heterostructure. The lattice constant of the triple-layered 2D-metal/2D-semiconductors heterostructure is calculated to be 5.04 Å. Thus, the lattice mismatch between GR and the MoSi_2_N_4_/MoGe_2_N_4_ heterobilayer is 2.38%, which is reasonably small and does not affect the main results. The interlayer spacings between the GR, MoSi_2_N_4_ and MoGe_2_N_4_ are calculated and listed in Table 2. For the GR/MoSi_2_N_4_/MoGe_2_N_4_, the interlayer spacings *D*_1_, *D*_2_ and *D*_3_ are calculated to be 3.58, 13.80 and 3.33 Å, respectively. These values indicate that the interactions between the GR, MoSi_2_N_4_ and MoGe_2_N_4_ are weak. In addition, we can see that the interlayer spacings of 3.33 Å and 3.58 Å are comparable with those in other 2D-layered heterostructures, which have typical vdW forces.^34–37^ This finding suggests that the GR, MoSi_2_N_4_ and MoGe_2_N_4_ layers in their corresponding heterostructures are bonded together *via* weak vdW interactions. It should be noted that the systems that are characterized by the vdW interactions are feasible and thus they can be easily fabricated in experiments using several common strategies, such as mechanical exfoliation^38^ and chemical vapor deposition (CVD).^39^ Furthermore, considering the MoSi_2_N_4_/GR/MoGe_2_N_4_ and MoSi_2_N_4_/MoGe_2_N_4_/GR heterostructures, we also find that they are characterized by weak vdW forces. Furthermore, in order to guarantee stability, we also perform the *ab initio* molecular dynamics simulations of such heterostructures at room temperature of 300 K, as depicted in Fig. 6. We find that the heterostructures are stable at 300 K room temperature.

**Table 2:** Calculated interlayer spacings between GR and MoSi_2_N_4_ (*D*_1_), GR and MoGe_2_N_4_ (*D*_2_) and between MoSi_2_N_4_ and MoGe_2_N_4_ (*D*_3_), binding energy, Schottky barriers, band gap and contact types of vdW heterostructures, forming between the GR, MoSi_2_N_4_ and MoGe_2_N_4_ monolayers

Heterostructures	Interlayer spacings			*E*_b_, meV per C atom	*Φ*_n_, eV	*Φ*_p_, eV	*E* ^GR^_g_, meV	Contact types
	*D*_1_, Å	*D*_2_, Å	*D*_3_, Å					
GR/MoSi_2_N_4_ (ref. 27)	3.16	—	—	−49.14	1.49	0.96	17	p-type ShC
GR/MoGe_2_N_4_ (ref. 27)	—	3.38	—	−24.86	0.63	1.54	14	n-type ShC
GR/MoSi_2_N_4_/MoGe_2_N_4_	3.58	13.80	3.33	−67.66	0.33	0.61	42	n-type ShC
MoSi_2_N_4_/GR/MoGe_2_N_4_	3.70	3.67	7.37	−55.73	0.43	0.41	46	p-type ShC
MoSi_2_N_4_/MoGe_2_N_4_/GR	14.23	3.31	3.45	−65.35	0.47	0.46	45	p-type ShC

The projected band structures of the heterostructures combined between the GR and MoSi_2_N_4_/MoGe_2_N_4_ heterobilayer are depicted in Fig. 5(d–f). Red and green circles represent the contributions of the GR and MoSi_2_N_4_/MoGe_2_N_4_ layers, respectively. One can observe that the electronic band structures of these graphene-based heterostructures seem to be a combination between those of the constituent GR and MoSi_2_N_4_/MoGe_2_N_4_ heterobilayer. A Dirac cone with linear dispersion of the GR layer is well preserved and located at the *M* point. The physical mechanism of the shift in Dirac point from the *K* to *M* point is due to the size effects in the GR supercell.^40^ The semiconducting feature of the MoSi_2_N_4_/MoGe_2_N_4_ heterobilayer is also maintained. The band gap of the MoSi_2_N_4_/MoGe_2_N_4_ heterobilayer contacted to the GR layer is about 0.84 eV, which is smaller than that of the freestanding MoSi_2_N_4_/MoGe_2_N_4_ heterobilayer. This finding suggests that the contact between GR and the MoSi_2_N_4_/MoGe_2_N_4_ heterobilayer tends to reduce the band gap of the MoSi_2_N_4_/MoGe_2_N_4_ heterobilayer semiconductor. This reduction gives rise to an enhancement in optical absorption of graphene-based heterostructures. Furthermore, the MoSi_2_N_4_/MoGe_2_N_4_ heterobilayer contacted with the GR layer possesses a direct band gap semiconductor with both the VBM and CBM at the *Γ* point, as depicted in Fig. 5(d–f). The nature of such a transformation from an indirect to direct band gap in the MoSi_2_N_4_/MoGe_2_N_4_ heterobilayer is due to a band folding effect. More interestingly, when GR contacts the MoSi_2_N_4_/MoGe_2_N_4_ heterobilayer, a band gap of about (17–46) meV is opened in the GR layer. It is clear that the mechanism of this band gap opening in the GR layer is due to symmetry breaking of the A and B sub-lattices in GR. This appearance was also observed in other GR-based heterostructures.^35–37^

**Fig. 7:**
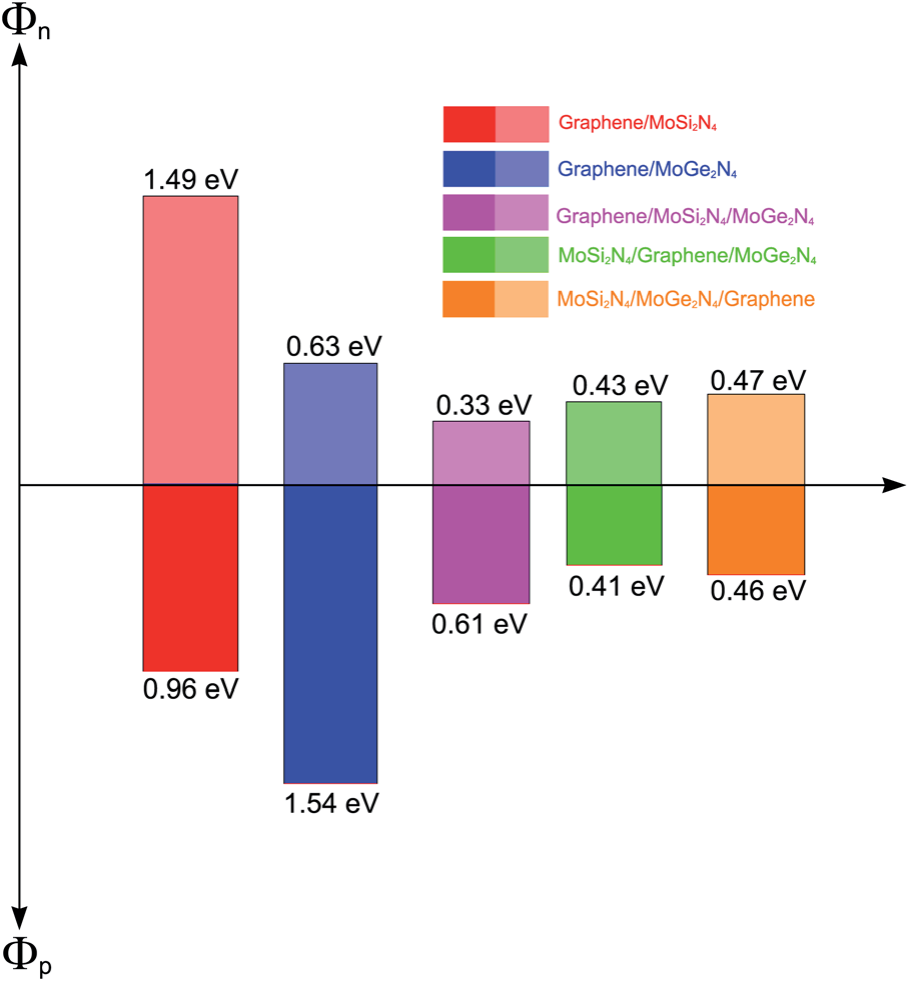
Histogram of the Schottky barrier height of the vdW heterostructures formed between graphene, MoSi_2_N_4_ and MoGe_2_N_4_ monolayers.

More interestingly, the contact between metallic GR and the semiconducting MoSi_2_N_4_/MoGe_2_N_4_ heterobilayer gives rise to the formation of a 2D metal/2D semiconductor interface. For the 2D metal/2D semiconductor interface, depending on the position of the Fermi level of metallic GR with respect to the band edges of the semiconductor, there is the formation of either Schottky contact (ShC) or Ohmic contact (OhC). One can find from the band structures in Fig. 5 that the Fermi level of the GR layer lies in the band gap region of the MoSi_2_N_4_/MoGe_2_N_4_ semiconductor, thus suggesting the formation of the ShC type in all three corresponding GR-based heterostructures. In addition, the Fermi level pinning is still weak in the heterostructures that are characterized by the weak vdW interactions. Therefore, we determine the contact barriers of such heterostructures using the Schottky–Mott rule.^41^ The n-type and p-type ShC, respectively, can be defined as *Φ*_n_ = *E*_C_ − *E*_F_ and *Φ*_p_ = *E*_F_ − *E*_V_, where *E*_C_, *E*_V_ and *E*_F_, respectively, are the CBM, VBM and Fermi level of the GR-based heterostructure. The calculated *Φ*_n_ and *Φ*_p_ of the GR/MoGe_2_N_4_/MoSi_2_N_4_, MoGe_2_N_4_/GR/MoSi_2_N_4_ and MoGe_2_N_4_/MoSi_2_N_4_/GR heterostructures are listed in Table 2. We find that the *Φ*_n_ in the GR/MoGe_2_N_4_/MoSi_2_N_4_ heterostructure is still smaller than the *Φ*_p_, indicating that it forms an n-type ShC at the equilibrium state. Whereas, for the MoGe_2_N_4_/GR/MoSi_2_N_4_ and MoGe_2_N_4_/MoSi_2_N_4_/GR heterostructures, the *Φ*_n_ is higher that the *Φ*_p_, implying that they form the p-type ShC. The *Φ*_n_ of the MoGe_2_N_4_/GR/MoSi_2_N_4_ heterostructure is calculated to be 0.33 eV, which is smaller than that of the GR/MoGe_2_N_4_/MoSi_2_N_4_ and MoGe_2_N_4_/MoSi_2_N_4_/GR heterostructures, as illustrated in Fig. 7. The lower the contact barrier, the better the device performance. Intriguingly, the contact barriers in the GR contacted MoGe_2_N_4_/MoSi_2_N_4_ heterobilayer are significantly smaller than those in the GR contacted with MoGe_2_N_4_ and MoSi_2_N_4_ monolayers, as listed in Table 2. This suggests that the GR/MoGe_2_N_4_/MoSi_2_N_4_ heterostructure offers an effective pathway to reduce the Schottky barrier, which is highly beneficial for improving the charge injection efficiency of contact heterostructures.

**Fig. 8:**
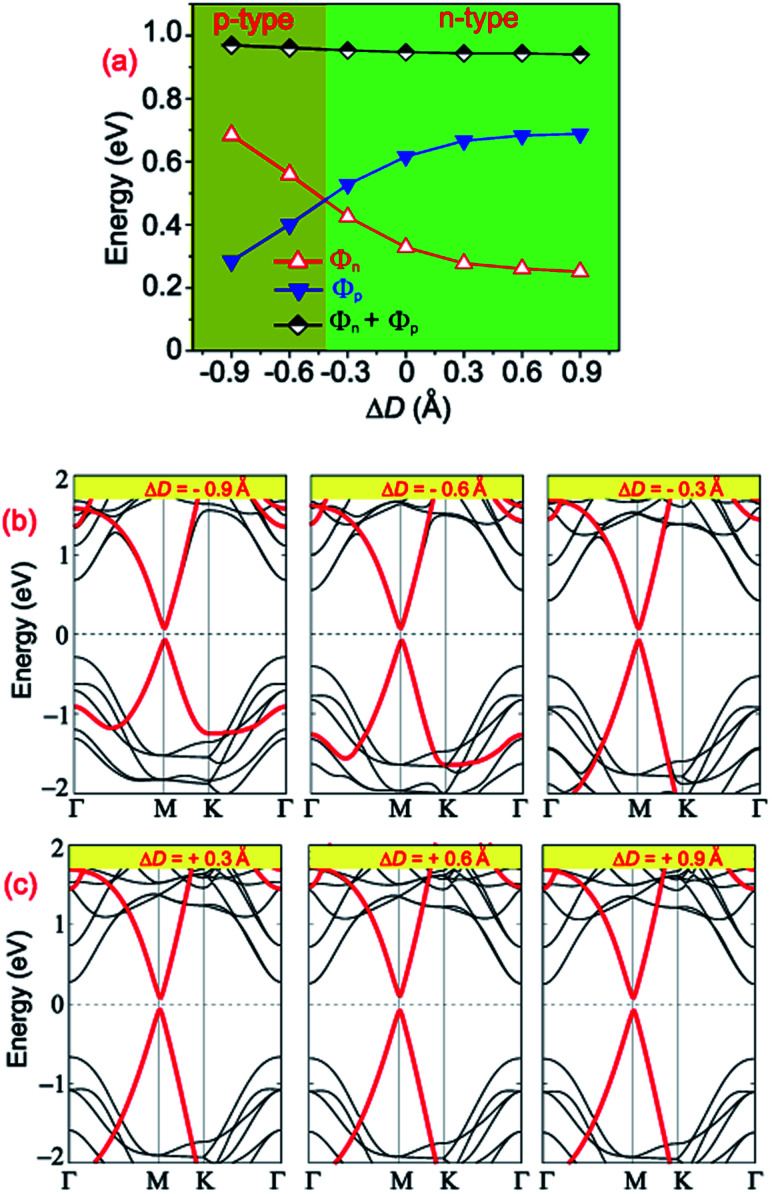
(a) The variation of the Schottky barriers of the GR/MoGe_2_N_4_/MoSi_2_N_4_ heterostructure as a function of the interlayer couplings. Projected band structures of the GR/MoGe_2_N_4_/MoSi_2_N_4_ heterostructure under (b) compressive and (c) tensile strains.

The tuning of the contact barriers and contact types in GR-based vdW heterostructures is an important factor of nanodevices based on the GR heterostructures. Controlling the interlayer coupling through stacking is one of the most efficient strategies in tuning both the contact barriers and contact types of GR-based heterostructures. The interlayer coupling can be manipulated by changing the interlayer spacings between the GR and semiconductors in their corresponding heterostructures. One can observe from Table 2 that the GR/MoGe_2_N_4_/MoSi_2_N_4_ has the lowest binding energy of −67.66 meV per C atom in comparison with that of other configurations, as it is the most stable configuration. Therefore, the effects of the interlayer couplings on the contact barriers and contact types of the GR/MoGe_2_N_4_/MoSi_2_N_4_ heterostructure are investigated as an illustrative example. The projected band structures of the GR/MoGe_2_N_4_/MoSi_2_N_4_ heterostructure as well as the variation in the contact barriers as functions of the interlayer couplings are displayed in Fig. 8. Red and black lines represent the contributions of the metallic GR and semiconducting MoGe_2_N_4_/MoSi_2_N_4_ heterobilayer, respectively.

Interestingly, we find that changing interlayer couplings gives rise to a change not only in the Schottky barriers but also in the contact types of the GR/MoGe_2_N_4_/MoSi_2_N_4_ heterostructure. The changes in the Schottky barriers and contact types in the GR/MoGe_2_N_4_/MoSi_2_N_4_ heterostructure are illustrated in Fig. 8(a). The *Φ*_n_ decreases with increasing interlayer spacing, while the *Φ*_p_ increases accordingly. On the contrary, the *Φ*_n_ increases with decreasing the interlayer spacing, while the *Φ*_p_ decreases accordingly. When the Δ*D* is smaller than −0.45 Å, the *Φ*_n_ becomes larger than the *Φ*_p_, leading to the transition from n-type ShC to p-type. The physical mechanism of such transitions can be described by analyzing the change in the Fermi level relative to the band edges of the semiconducting MoGe_2_N_4_/MoSi_2_N_4_ heterobilayer. With the applications of the compressive strain, *i.e.* Δ*D* < 0, the Fermi level of graphene moves towards the VBM of the semiconducting MoGe_2_N_4_/MoSi_2_N_4_ heterobilayer, thus resulting in an/a increase/decrease in the *Φ*_n_/*Φ*_p_. On the other hand, with the application of the tensile strain, *i.e.* Δ*D* > 0, the Fermi level of graphene shifts upwards from the VBM to the CBM of the semiconducting MoGe_2_N_4_/MoSi_2_N_4_ heterobilayer. Thus, the *Φ*_n_ is decreased and the *Φ*_p_ is increased accordingly. In this case, the heterostructure remains as n-type ShC. It can be concluded that with the application of different strains, both the Schottky barriers and contact types in the GR/MoGe_2_N_4_/MoSi_2_N_4_ heterostructure can be manipulated.

## 4. Conclusions

In summary, we have investigated the structural and contact types in a 2D vdW heterobilayer between MoGe_2_N_4_ and MoSi_2_N_4_ monolayers, as well as in the presence of electrical graphene. We found that the MoGe_2_N_4_/MoSi_2_N_4_ heterobilayer forms type-II band alignment, which effectively promotes the separation of electrons and holes, and provides an opportunity for further electrons and holes. Interestingly, the insertion of the electrical graphene contact to a MoGe_2_N_4_/MoSi_2_N_4_ heterobilayer gives rise to the formation of a metal/semiconductor contact, which is characterized by the contact type barriers. Depending on the graphene position relative to the MoGe_2_N_4_/MoSi_2_N_4_ heterobilayer, the graphene-based heterostructure can form either an n-type or p-type Schottky contact. The graphene/MoGe_2_N_4_/MoSi_2_N_4_ heterostructure exhibits an n-type Schottky contact, whereas the MoGe_2_N_4_/GR/MoSi_2_N_4_ and MoGe_2_N_4_/MoSi_2_N_4_/GR heterostructures form a p-type one. Intriguingly, the contact barriers in the GR contacted MoGe_2_N_4_/MoSi_2_N_4_ heterobilayer are significantly smaller than those in the GR contacted MoGe_2_N_4_ and MoSi_2_N_4_ monolayers, suggesting that the GR/MoGe_2_N_4_/MoSi_2_N_4_ heterostructure offers an effective pathway to reduce the Schottky barrier, which is highly beneficial for improving the charge injection efficiency of the contact heterostructures. More interesting, by controlling the interlayer coupling through stacking, both the Schottky barriers and contact types in the GR/MoGe_2_N_4_/MoSi_2_N_4_ heterostructure can be manipulated.

## Conflicts of interest

There are no conflicts to declare.

## Supplementary Material
